# Implementation of research, education and leadership placements into Operating Department Practitioner training: A 4-pillar practice-based learning approach

**DOI:** 10.1177/17504589241276743

**Published:** 2024-10-13

**Authors:** Victoria Cadman, Helen Batty, Jennifer Law

**Affiliations:** Sheffield Hallam University, Sheffield, UK

**Keywords:** ODP, Operating Department Practice, Placement, Research, Education, Leadership

## Abstract

Practice-based learning has traditionally focused on clinical practice in pre-registration courses. However, recent national strategies emphasise the importance of incorporation of all four pillars, clinical, education, leadership and research, into practice-based learning (placements). This article details the introduction of practice-based learning in research, education and leadership alongside clinical placements for BSc Operating Department Practice students at Sheffield Hallam University. It provides insights into the benefits of this approach, outlines the components of each placement with examples of completed projects, shares feedback from students and practice partners and addresses challenges. The authors advocate for adoption of this approach across all pre-registration Operating Department Practice courses, to ensure a workforce capable of meeting evolving health care needs and driving the Operating Department Practice profession forwards.

## Introduction

Practice-based learning (PBL) is an integral component within pre-registration training provision of health care professionals. Traditional approaches deliver theoretical knowledge through academic modules, with PBL (placements) usually focused on clinical placements. Our institution, Sheffield Hallam University (SHU), has introduced an innovative strategy to include placements with research, education and leadership. This enhanced approach bridges this gap between theory and practice, giving students a more comprehensive perspective of health care, with the aim of improving their practice, enhancing employability and raising their career aspirations. This discussion outlines the development and implementation of this new approach presenting examples of projects undertaken by students, discusses the feedback to date and highlights the positive impact on students’ professional development and readiness for the challenges within future health care.

## Background

The four pillars of practice, clinical, education, leadership and research, were introduced as part of a multi-professional framework to underpin the development of advanced practice within health care professions (Health Education England (HEE) 2017).These pillars are valuable for all levels of qualified practice to provide structure for professionals to assess their progress and developmental needs as they plan their career route. The focus of development will vary depending on the scope, role and context of their practice, and for many practitioners, this would most likely lie within the clinical pillar. However, development across all four pillars is considered essential for a well-rounded health care professional capable of thriving within the changing National Health Service (NHS) landscape and is a requirement for those progressing to advanced practitioner roles (HEE 2017).

While Operating Department Practitioners (ODPs) are accepted into several areas of practice relatively easily, many barriers still exist for those wanting to progress into non-traditional routes. This is often due to other professions’ unfamiliarity or perceptions of the role and/or training programme. This leads to a lack of opportunity to gain experience and insight into particular career paths, and few role models to provide guidance and mentoring in overcoming barriers such as challenging job descriptions so that ODPs can apply. Consequently, while some ODPs have successfully progressed into non-traditional and senior health care pathways, the profession faces challenges characterised by skills gaps arising from generational and educational factors. While formal research evidence supporting these observations is lacking, the perceived disparity offers insights into the workforce profile of ODPs. Currently, 54% of ODPs hold positions at Band 5, 33% are Band 6, with only 11% in roles at Band 7 or higher. A small number are reported below Band 5. This is in stark contrast to the nursing profession where 41% are at Band 5, with 24% at Band 7 and above, and allied health professionals (AHPs), where only 21% are at Band 5, with 45% at Band 6 and 31% beyond this level (HEE 2023). Specifically, areas such as leadership, supervision, assessment of learners and research have been identified for additional exploration to optimise the ODP workforce (HEE 2023). These gaps, highlighted by the current underrepresentation of ODPs in diverse roles, limit opportunities to inspire the next generation.

Recent national strategies advocate for PBL for pre-registration learners to include all four pillars of practice to ensure graduates are capable of high-quality patient-centred care and able to meet the demands of future health care (NHS England 2020, HEE 2022, NHS England 2022). The introduction of PBL guidelines (Chartered Society of Physiotherapy & Royal College of Occupational Therapy (CSP & RCOT) 2023), endorsed by the College of Operating Department Practitioners (CODP), includes the requirement for PBL to take place across all areas, pillars and levels of practice, yet the emphasis for PBL within the pre-registration curricula is within the clinical pillar (CODP 2018). PBL in research, education and leadership can bridge the gap between theory and practice ensuring graduates have the skills and capabilities needed to deliver best practice. We need our future ODPs to be our researchers, educators and leaders of the future.

Given these national strategies, as a pre-registration education provider, we wanted to diversify the career aspirations of our students by exposing them to placement experiences related to other career pathways. This would not only demonstrate that it is possible for ODPs to undertake such roles, but would allow development of other skills and adaptability outside of the clinical environment. This would, in turn, be beneficial for enhanced knowledge and understanding for their clinical practice, wider knowledge of health care provision and help drive the ODP profession forwards.

## Implementation of placements

PBL in research, education and leadership had already been introduced within other AHP pre-registration courses at our institution. However, these were all within professions where assessment was based on achieving overall learning outcomes rather than specific clinical competencies. In contrast, the ODP programme requires students to demonstrate competency of a specific set of clinical skills relating to the particular practice area and professional attributes. Consequently, allocating ODP students to this type of placement would reduce their time for achieving clinical competencies and subsequently impact on the clinical time available to them for successful completion of the course. Therefore, a format had to be found that meant that *all* students would undertake this new form of placement ensuring an equitable experience and that no student was disadvantaged by reduced clinical time.

A review of the academic calendar identified that it would be possible to adjust our placement pattern to incorporate a 4-week research, education or leadership placement for every student. As a result, every student received an equitable distribution of clinical time, as well as maximised opportunities for the development of research, education, leadership and associated skills. This in turn would enhance the employability of students as they had different skills to offer other than the traditional clinical placement experience. Feedback from graduates from other AHP professions who had completed this type of placement highlighted that discussing these placements and their achievements at interview was a unique selling point and employers were impressed with their enhanced skills outside of their clinical skills.

Placement blocks were provided on a rotational basis in four blocks across the academic year meaning 75% of students remained in clinical practice areas, with 25% in alternative settings. This small decrease in student numbers in clinical settings had the added benefit of supporting departments to be able to deliver high-quality learning experiences for students despite the reduced surgical activity resulting from the ongoing impact of the COVID-19 pandemic. Enquiries and initial planning for potential placement providers and projects were prepared during the summer prior to implementation. While approaches to clinical partners were being made, it was possible to run several projects within our institution, either with members of staff in the ODP team, wider department and associated research centre.

A format and guidance for the components of each placement were produced, based on recommendations already in place within the institution across other AHPs. This consisted of:

- A multi-student approach (a minimum of 2–4 students in a group working together).
- A recommended model of supervision.
- Common activities such as induction, progress reviews, recommended reading and self-directed learning activities.
- A final presentation.

Other activities were dependent on the placement area and were facilitated by colleagues throughout relevant departments. Placements were face to face or a hybrid model depending on the area. Further details of example projects are provided in a later section. Examples of what each placement type may include are shown in Table 1.

**Table 1:** What is included in research, education and leadership PBL

Research	Education	Leadership
Contribute to the design of a study	Design teaching sessions	Develop a project idea
Write a literature review	Create teaching resources	Manage and complete a project
Identify or apply for funding for a research project	Support teaching/staff development sessions	Attend leadership meetings with other professionals
Prepare ethics proposals	Deliver teaching sessions, face-to-face and online	Peer support with other students
Data collection	Support students/staff	Work as a team
Data analysis	Work within the team as a student lecturer/teacher	Work independently
Interpreting the data and drawing conclusions	Complete online training package on adult learning theories/teaching principles	Complete leadership training – Edward Jenner
Writing up a study		
Research training – Good clinical practice training and/or data security awareness course		

A change management process was introduced for students and practice partners. Our practice partners were invited to attend a face-to-face meeting where we presented the rationale for the introduction of the placements, the proposed timetable and outlined plans for projects students would be undertaking. We created a screencast recording of this information which was uploaded to the ODP placement external website so that the information could be accessed widely. The information was also shared via email, together with an informative video on the four pillars of practice produced by HEE (2021). We recognised the importance of reaching all clinical educators who support our students in practice, so we also discussed the placements during on-site visits and educator updates. Students were initially informed of the introduction of the new type of placement at the end of their second year as part of their transition activity so they were aware of what their final year of study and placement would look like. On student’s return to the course after the summer vacation, their preparation for the placement was undertaken. This included on-campus and online sessions during the first week of the academic year and detailed face-to-face induction sessions at the beginning of every placement block. Further information about the rationale and implementation of the placements was also added to the student online teaching platform, Blackboard. This multi-faceted approach aimed to ensure that all relevant parties were well-informed and equipped with the necessary details for successful implementation.

As this was the first time we had included this type of placement block, students were asked to indicate a preference for the type of placement they would prefer and this was accommodated as much as possible. We wanted these PBL experiences to be as inclusive as possible for our students.

## Assessment

The 4-week placement block is an integral part of students’ placement module which runs throughout the academic year and is assessed on a pass/fail basis. During the 4 weeks, students are assessed on their professional competencies and are required to upload evidence to their online portfolio. If the block is not completed to a satisfactory standard, students must complete a reassessment placement to achieve a pass.

## Example projects

All placements incorporated a variety of activities and ways of working that students had to adapt to and thus demonstrate their professional skills in a way that they were not used to on clinical placements.

### Leadership placements

During leadership-focused placements, students completed a project as a group. They engaged in the Edward Jenner Programme level 0, and, if time permitted, they progressed to level 1. This online programme provides fundamental, underpinning leadership theory, enhancing their understanding of key leadership principles. Given the importance of this learning material, the decision was made to incorporate it into the initial stage of all leadership placements ensuring students gained early insight and understanding as they embarked on their placement. Valuable projects have been successfully completed leading to real impact for both practice partners and students. For example:

- *Inclusivity in practice*: Students developed a website of resources and guidance for practice educators and fellow students with the aim of improving student experience on clinical placement. This project focussed initially on race and religion with a subsequent group contributing to include disabilities and neurodivergence. The project required extensive research of the literature, data gathering from stakeholders, attendance at senior leadership meetings and project management skills to ensure timely completion.
- *Creation of an immersive theatre experience*: Students created an immersive virtual reality (VR) theatre experience for first-year students which aimed to alleviate anxiety and enhance knowledge of clinical placement areas and equipment prior to clinical placement. This project involved in-depth research on VR, identification of suitable locations and equipment for inclusion in the VR tour, interviewing stakeholders, collaboration with technical, clinical and academic teams and effective project management skills to meet the project’s timeframe.

### Education placements

Education placements were provided and supported by staff within the ODP team. As well as initial reading and activities to enhance their knowledge and understanding of adult learning theories, students observed and shadowed teaching staff in a variety of teaching activities for students at levels 4 and 5 (first and second years). Students were integrated into the teaching team, attending team meetings and contributing to course discussions, providing valuable insights and ideas. Any confidential student-related discussions were left until the latter part of meetings after students had left. This format provided students with the experience of most aspects of formal teaching and related activities within the delivery of the ODP programme. Students then applied their learning and knowledge to develop and produce a variety of learning resources and deliver these to first- and second-year students. These included digital learning packages, clinical skills sessions for academic modules and simulated placement, and peer assessment support for Objective Structured Clinical Examinations (OSCEs).

### Research placements

Research placements were conducted by staff within SHU’s Advanced Wellbeing and Research Centre (AWRC) where students worked alongside researchers on the Active Wait Project. This research project, developed in collaboration with the Musculoskeletal Care Group at Sheffield Teaching Hospitals NHS Foundation Trust, explores enabling people on the waiting list for hip and knee replacement to remain active and be prepared for surgery (SHU 2024). During the students’ 4-week placement, they attended preoperative clinics and ‘Joint School’ sessions gathering data from service users. They were actively involved in professional meetings with clinicians and researchers to co-create a 12-week digital course designed to provide information and an exercise programme for those awaiting surgery (Sheffield Hallam University Advanced Wellbeing Research Centre & Sheffield Teaching Hospitals NHS Foundation Trust 2023).

## Feedback

**Figure 1:**
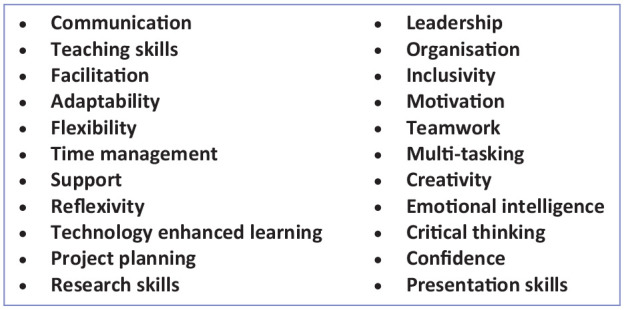
Enhanced skills

Students initially struggled to see the relevance of the placements to their future clinical practice and were worried they were missing valuable clinical experience. However, following their placements, they had changed their views considerably stating they would recommend these placements to other students. Their experience had opened their eyes to the many different career paths which are accessible to ODPs and which they had not known about previously. They recognised the value of the skills they had learnt to their employability and their future clinical practice. Some of the skills the students believed they had enhanced are shown in Figure 1, all of which contribute significantly to effective clinical practice.

A recent graduate stated ‘*My leadership placement played a pivotal role in equipping me with the skills and confidence to take opportunities I might not previously have considered*’. This includes working in areas she had not previously worked in, taking an active involvement in educating students and engaging in service improvement projects. The experience also empowered her to apply for a master’s degree, and to write up her dissertation for publication.

Staff within the university, both within the ODP and broader institution, along with partners who facilitated the placements, have really appreciated the valuable insights, thoughts and ideas that students contributed to meetings and workshops as well as the high-quality work the students achieved which would not have happened otherwise. Feedback from staff in clinical practice has been mixed, mainly as a result of misconceptions regarding the placements. To help address some of these concerns, we invited some students to present the work they had completed at one of the clinical partner meetings which was positively received. Further work to address feedback is outlined in later sections.

## Barriers and facilitators

Managing the perceptions and expectations of both students and clinical staff and promoting the value of this form of placement was the most challenging aspect of implementation. A change management process was required to educate both students and clinical partners of the rationale and national agendas around the four pillars of practice and the benefits to clinical practice and employability skills. It helped that these placements had already been implemented in other professions and a website of information and resources was already available which students could access prior to their placement. Colleagues both within our institution and from external organisations were highly supportive. They welcomed students to meetings and workshops, as well as providing bespoke sessions, for example, project management, inclusive leadership, professionalism within the workplace and clinical research delivery. Students gained in-depth understanding of relevant systems, processes and organisations, learnt new skills from senior leaders and gained experience with a range of different AHPs. They were offered unique opportunities which generated discussion across the cohort, highlighting the benefits of these placements. This reduced negative perceptions among the student body. However, there remains some scepticism from clinical colleagues as to the perceived value of these placements. A change in culture is necessary. We are actively addressing this through regular mentor updates and by encouraging students to discuss their work openly during their clinical placements to highlight the value and benefits.

## Recommendations

During the implementation and the first year of placements, we listened to students, staff and external partners and as a result have made adjustments. This has included amendments to the supporting paperwork for the placement, increased preparation of students, and raising and maintaining the profile and value of the placement with practice partners. We are currently working with our external partners to increase these placements within clinical departments, meaning that projects and research currently difficult to undertake due to the pressures on service, can be undertaken by our students for the benefit of service delivery. Conversations are ongoing so that these placements continually evolve to reflect the changing landscape of both the profession and health care and we envisage that this will continue to provide a relevant placement experience for students. We feel that this form of placement is of significant value and should be considered as being integral to all ODP training programmes. This will ensure graduates are capable of working in the ever-changing health care environment as well as promoting and exposing students to other career roles and opportunities. These placement experiences support recommendations made by HEE (2023) which reported on the ODP workforce and the recent AHP Practice Learning Principles (CSP & RCOT 2023). Embedding the expectation that ODPs work across all four pillars of practice during training is an essential step forward in changing perceptions and enabling opportunities for individual and workforce development of the profession. As with any innovation, research data need to be gathered to inform, and we aim to complete formal evaluation on this placement experience in due course.

## Conclusion

PBL in research, education and leadership has been successfully implemented for all pre-registration BSc ODP learners at SHU. Embedding these placements within all pre-registration ODP programmes nationally is essential to develop a workforce capable of best practice and able to drive the profession forwards. If we do this, our future ODPs will have the knowledge, skills and resilience required to deliver best practice, apply research to practice, lead and manage change, educate the future workforce and champion the ongoing progression of our profession.
